# Assessing strategic, tactical, and operational decision-making and risk in a livestock production chain through experimental simulation platforms

**DOI:** 10.3389/fvets.2022.962788

**Published:** 2022-10-20

**Authors:** Christopher Koliba, Scott C. Merrill, Asim Zia, Gabriela Bucini, Eric Clark, Trisha R. Shrum, Serge Wiltshire, Julia M. Smith

**Affiliations:** ^1^Social Ecological Gaming and Simulation Lab, School of Public Administration and Affairs, University of Kansas, Lawrence, KS, United States; ^2^Social Ecological Gaming and Simulation (SEGS) Lab, Plant and Soil Science Department, Gund Institute for Environment, University of Vermont, Burlington, VT, United States; ^3^Social Ecological Gaming and Simulation (SEGS) Lab, Plant and Soil Science Department, University of Vermont, Burlington, VT, United States; ^4^Social Ecological Gaming and Simulation (SEGS) Lab, Community Development & Applied Economics Department, University of Vermont, Burlington, VT, United States; ^5^Plant Biology Department, Food Systems Research Center, University of Vermont, Burlington, VT, United States; ^6^Social Ecological Gaming and Simulation (SEGS) Lab, Animal and Veterinary Sciences Department, University of Vermont, Burlington, VT, United States

**Keywords:** biosecurity, compliance, scaling, micro-macro, risk perception, nudges

## Abstract

This paper provides a research summary of a series of serious games and simulations that form the basis of an experimental platform for the study of human decision-making and behavior associated with biosecurity across complex livestock production chains. This platform is the first of its kind to address the challenges associated with scaling micro-behavior of biosecurity decision-making to macro-patterns of disease spread across strategic, tactical and operational levels, capturing the roles that facility managers and front-line workers play in making biosecurity decisions under risk and uncertainty. Informational and incentive treatments are tested within each game and simulation. Behavioral theories are used to explain these findings. Results from serious games in the form of behavioral probability distributions are then used to simulate disease incidence and spread across a complex production chain, demonstrating how micro-level behaviors contribute to larger macro-level patterns. In the case of this study, the propensity to adopt micro-level biosecurity practices are applied to a network percolation disease spread model. By presenting the suite of companion models of behavior and disease spread we are able to capture scaling dynamics of complex systems, and in the process, better understand how individual behaviors impact whole systems.

## Introduction

Computational modeling, digital serious gaming, and simulation tools can provide a better understanding of how the percolation of individual behaviors manifests itself across networks of heterogeneous actors to produce specific systemic outputs and outcomes. The spread of disease across human systems, like during the global COVID-19 pandemic, and increasingly, systems of domesticated livestock, provides an opportunity to look at the relationship between individuals' micro-level behaviors operating across different levels of operational, tactical or strategic action and their implications for more macro-scale outcomes. This research summary provides an overview of an experimental simulation platform designed to study the relationship between micro-level individual behavior and macro-level outcomes within disease spread scenarios. Researchers are increasingly using computational modeling and serious gaming to consider and study systems across micro and macro levels. Understanding scalar dynamics can lead to predicting and mitigating disease risk transmission by helping to align private and public costs and beneifts associated with biosecurity, and anticipate how decisions made at operational scales can impact the wider spread of disease among livestock populations.

To capture the micro-level roles and behaviors of individual human actors spanning the continuum of front-line workers, managers and executive leaders, a series of serious games that operate at different strategic-tactical-operational levels was developed. The results from these games are used to calibrate whole systems models of a livestock production chain using an agent-based modeling (ABMs) approach to demonstrate how behavioral nudges at different levels of operational and tactical action scale can form specific micro- and macro-level behavior relating to mitigating disease transmission. The approach and findings can have a bearing on developing a coordinated policy response to highly contagious disease spread events.

This research summary begins with clarifying a critical question relating to human agency and macro patterns, and specifically distinguishes between actors operating at different levels of strategic, tactical and operational collective action, contributing to our greater understanding of micro-macro scaling phenomena in complex social systems. We then introduce the context of this particular study, livestock biosecurity, and define a very specific set of considerations that are confronting policy makers, executive leaders, managers and front-line workers in this area. The methods used to build our experimental simulation platform are then discussed. The paper walks the reader through each gaming and modeling component, providing high level summaries of findings from each. The paper concludes by returning to the larger questions of micro-macro scaling and the contributions to theory and methodological development that this particular body of work has contributed to.

## Materials and methods

The growth in the depth of understanding critical behavioral biases such as bounded rationality, implicit bias, the role of framing and anchoring effects, etc. have expanded our understanding of how people make decisions and behave in specific policy and management settings (1). Donald Moynihan, discusses the differences between micro-level “nudges” (2) and macro-level policy and institutional “shoves” [(3), p. 3], and observes that recent research in behavioral public administration has tended to focus almost exclusively on the “smaller” questions of individual motivation and behavior at the exclusion of some of the bigger questions focusing at more macro levels, e.g., those activities that impact whole systems. Moynihan is concerned about the development of a “micro-macro schism,” much like those found between micro and macroeconomics and to a lesser extent micro and macro sociology (4). To overcome the rise of this micro-macro schism, Moynihan calls for integrating the findings from behavioral experiments into more aggregated models of organizational and institutional systems. Doing so may allow us to scale-up tactical, operational, and strategic micro behaviors of individuals to answer macro questions pertaining to organizational and network performance and ultimately better understand the roles of human behavior as drivers of policy implementation and policy failure. Conversely, we may also come to better understand how macro drivers scale-down to influence micro behaviors.

The challenges with understanding how micro-macro dynamics unfold in complex settings is often understood in terms of “micro-macro scaling” questions: How does micro behavior and decision-making scale-up to inform larger, macro systems of coordinated action? How do macro-scaled institutional norms, organizational cultures, institutional rules and policies scale-down to shape operational, tactical, and strategic micro behaviors? Livestock production systems can be understood as “complex adaptive systems” that possess emergent qualities that are driven by the internal, self-organizing features of the system (5). The charactersitics of emergence in these kind of systems can be understood as “bottom up” materializations of a myriad of indivudal acts of behavior that “add up” to form larger, larger system-level dynamics. In the instance of livestock production, the cumulative behaviors of thousands of farm workers will have a role to play in the extent which disease is spread. While other actors in the production chain, including farm managers, owners, regulators and service providers (veterinarians) also render decisions and behaviors that have emergent, bottom up impacts on the wider system.

When we consider the question of micro-macro scaling, the positionality of actors within an organizational or institutional structure very likely matters. Theories of leadership and management have tended to assert that any one person can exert authority and influence over the direction of organizations and networks. Those with the greatest influence are said to be the people in positions of strategic leadership roles (6–9). Good managers can execute their span of control and unity of command to link tactical decision-making to daily operations by seeking the compliance and cooperation of subordinates. Front-line workers who often serve at the “street-level” provide direct service and discretionary authority that impacts the performance of daily operations (10). The discretion of individuals operating across a strategic-tactical-operational continuum combine to shape the functioning and performance of organizations and networks of organizations. In turn, it has been noted how the collective norms, organizational cultures, institutional rules and policies found in organizations and institutions can shape and inform the behaviors of individuals (11). These macro-level properties can scale-down to the micro-level of individuals making strategic, tactical and operational decisions. By examining these processes, we can better understand how the discretion of different actors across different scales of decion-making not only influences the likelihood of biosecurity failures, but *should* influence the discretion of actors at the tactical and strategic scales. A better understanding and appreciation of how their own and others' discretion and actions increases or decreases the likelihood of disease spread.

**Figure 1:**
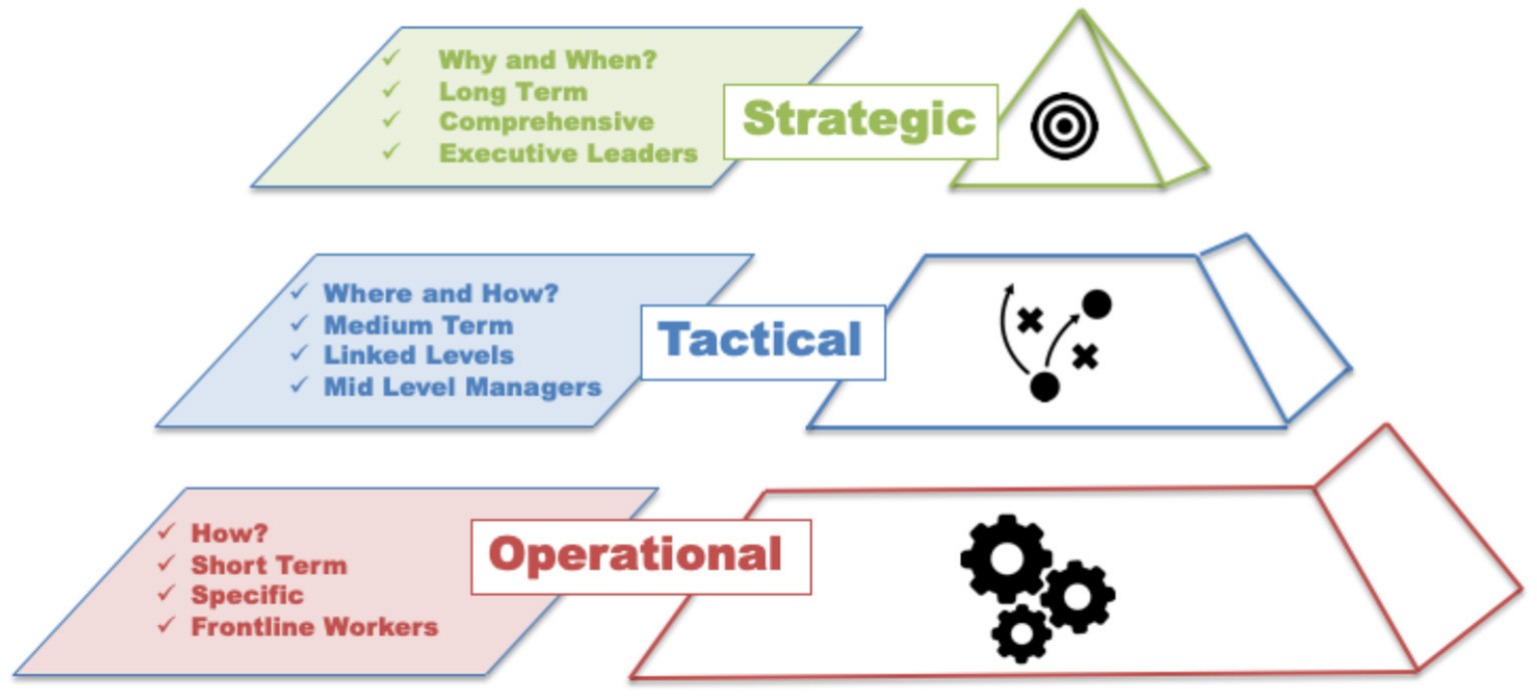
Scales of discretion: The STO framework.

In order to understand the impacts of micro-level individual discretion on larger levels of collective action, we must first clarify the architecture that has been implicit in those organizational theories that view organizations as systems that rely on the agency of specific actors assuming specific operational, tactical or strategic roles. Drawn from the distinctions between the levels of social system behavior (6, 7, 9, 12), a strategic-tactical-operational (STO) framework for understanding the scales of discretion across a given social system is presented here. Figure 1 provides an overview of this framework as a tiered system that is differentiated by the types of questions asked (Why and when? Where and how? How?), the temporality of those decisions (long to short term), the degree of comprehensiveness to specificity, and the roles and functions of decision makers and actors (from executive leaders to front-line workers).

Ackoff (13) first distinguished between tactical and strategic action within a systems context parsing the differences between short term matters of basic operation and longer term coordinated actions of linked services and levels. More recently, Loorbach (14) focused on the application of strategic, tactical and operational planning, tasks and decision making to transition management. He notes how change unfolds within and across complex systems and calls for prescriptive models that account for the heterogeneity of actors that include top down, market, collaborative and reflexive features [(14), p. 166].

Discrete studies and experimentation at each scale are needed to inform prescriptive “nudges” and other forms of inducements, sanctions and regulations. In the context of the studies summarized here, the focus is on nudges that come in the form of risk communication methods. The types of systems' level and impactful policy “shoves” (inducement, sanctions and regulations) that are likely needed to better mitigate systemic risk across entire systems of livestock production require the integration of data on human behavior across different scales of discretion to better understand how biosecurity protocols and practices may be enacted. Where possible, the integration of this data on the propensity of decisions made at the operational and tactical scales should inform the development of decision support tools to inform strategic planning at the most macro level (14, 15). The wicked problem of livestock biosecurity provides an excellent context to apply this approach.

### The research context: Livestock biosecurity

The focus of this particular study and application is on emerging and endemic livestock diseases of social and economic importance that can have profound policy implications, impacting food security, food safety, animal welfare, and domestic and international trade. Outbreaks of infectious animal diseases can lead to losses of millions of animals and billions of dollars. For example, in just 6 months, 50 million chickens and turkeys were killed in the U.S. during an outbreak of highly pathogenic avian influenza in 2017 (16) and almost 38 million birds in 2022 (17). This outbreak led to losses of over $3 billion in the U.S. poultry industry (18). These outbreaks can have potential negative environmental, health and economic consequences from mass disposal of animal products or carcasses, as well as the potential transmission of diseases to other species, including humans (19). Direct and indirect economic costs to producers, processors or packers send ripples into local, regional and national economies and may result in food insecurity domestically or abroad. We ignore the potential direct costs to humans stemming from zoonotic disease transfer (e.g., Ebola and coronaviruses), and focus on the costs associated with animal foodstock production. With the rise of industrial agriculture, the health considerations of livestock herds have been extended to the ability to raise, send, receive and sell healthy animals across the livestock production chain. Global markets and the internationalization of livestock trade, feed consumption and related farming resources have not only raised the stakes in livestock commodity markets economically, but have also opened livestock production chains to greater disease risk (20).

The Animal Disease Biosecurity Coordinated Agricultural Project (ADB-CAP) funded by the U.S. Department of Agriculture (USDA) was undertaken by the co-authors and developed on the assumption that micro-scale human decision-making and behaviors play significant roles in the introduction, spread, recognition, reporting and containment of new, macro, systems level emerging or foreign diseases. The goal of the ADB-CAP project was to facilitate the development and adoption of practices and policies that collectively reduce the impact of new, emerging and foreign diseases of livestock in the United States. Building off the success in ADB-CAP, our team is working on a successor project funded by USDA NIFA under NSF EEID program to predict livestock disease transmission dynamics under alternate biosecurity risk management interventions and behavioral responses of livestock producers. To effectively govern biosecurity risks, this requires an approach that takes into account the micro-scale behaviors of individual human actors in the production chain, and the more macro-level properties of the industry and policy structure, and disease spread characteristics. Whether viewed from the perspective of disaster preparedness or emergency management, behavior change or economic decision-making under uncertainty, the process of making biological risk management decisions to mitigate multiple disease threats is complex in part because it relies on the micro-scale decisions and behaviors of front-line workers, facility managers, production chain owners and policy makers. In turn, these decisions and behaviors essentially scale-up and impact the spread of disease across production chain networks.

For the purposes of the simulations and serious games summarized here, the disease threat of Porcine Epidemic Diarrhea virus (PEDv) to the swine industry in the United States was gamified and simulated. This disease is transmitted *via* a fecal to oral route through direct or indirect contact. PEDv is not zoonotic (meaning it does not transfer from animals to humans) in nature and the overall threat to the industry and individual farmers is, to date, manageable. However, systems level outbreaks of PEDv and other similar diseases have the potential to seriously compromise herd health as well as the livelihoods of many people throughout the production chain (21).

Risk mitigation of the livestock food supply is a concern of policy makers and government regulators across multiple levels of jurisdiction, and most particularly at the federal and state level. As such, animal health authorities operating at the federal and state levels are faced with decisions regarding if, how or when to uses incentives or regulations to mitigate livestock disease risk.

The main risk driving the system's behavior is risk of transmission of infectious material between animals and between animal production facilities and the inherent risk to ecomonic viablity. Operational risk management falls predominantly to front-line workers, whose compliance with biosecurity practices, such as shower-in/shower-out and “line of separation” protocols, is often the deciding factor as to whether particular animals within facilities get infected. Tactical risk assessment falls predominantly to the owners or managers of facilities who make the decision to implement specific biosecurity protocols. Strategic risk assessment generally falls to owners of production chains, policy makers and technical assistance providers. Their concerns center on the viability and vulnerability of large-scale operations and even the economic, social and environmental viability of entire industries.

The ability to capture the wide range of operational-tactical-strategic risk and decision-making factors that shape livestock biosecurity practices requires a systems approach to experimentation and modeling. To gather data on the full range of risk assessment and practices, a series of serious games operating at different scales of decision-making were designed. A “compliance” game focused on operational decisions of front-line workers (22). In a “protocol adoption” game, the focus is on tactical decisions of facility managers to adopt biosecurity protocols (23). Risk communication strategies are employed as treatments in both games. Prospect theory (24), risk aversion and incentive effects (25, 26), uncertainty aversion (27), and temporal discounting effects (28, 29) are used to explain the results in both games.

**Figure 2:**
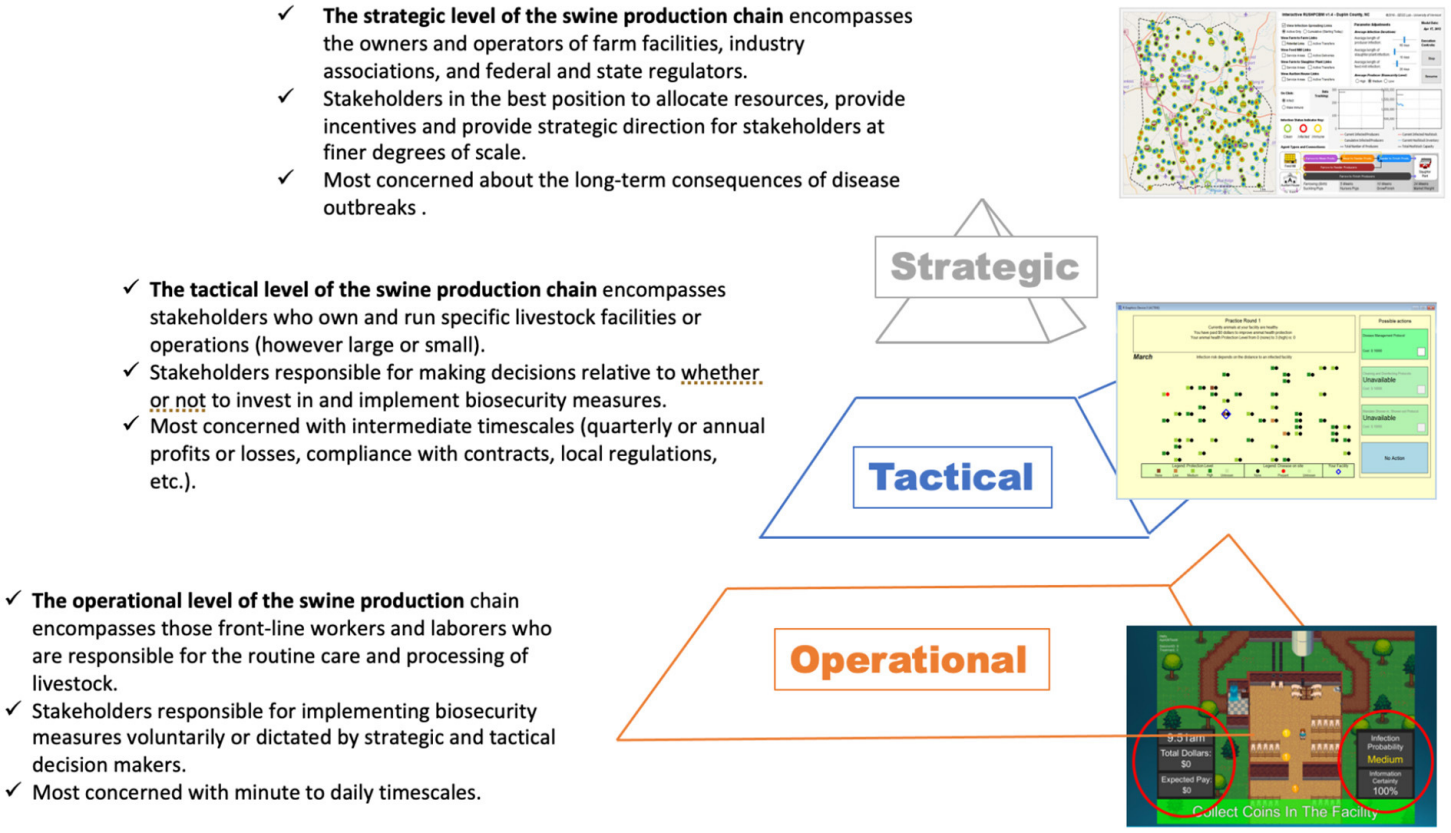
STO framework applied to swine production chain.

To simulate the role that operational and tactical behaviors play in the spread of disease, our team developed an agent-based model (30–32) that draws on network-level theories of diffusion (33) and percolation (34) to capture the spread of both disease and behaviors across complex production chain networks (31). A later version of the model simulated strategic decision making informed by risk aversion or risk-taking behavior pulled from prior game results” (32). Using the results of the gaming experiments to parameterize behavior within the simulation model, we are able to ascertain how the probability distributions of observed risk-taking and aversion at the operational and tactical scales can align with observed outbreak dynamics, and simulate outbreak scenarios with alternative risk-taking and aversion behaviors, such as those observed during serious game experimentation under aggressive communication strategy policies. In the process we are able to better understand the role that the behaviors of front-line workers and facility managers under alternate incentive and risk communication regimes contribute to the mitigation or promulgation of livestock disease. Using this understanding, we can ascertain the efficacy of policy nudges on disease spread phenomena. Figure 2 provides an overview of the relationship between the two games operating at the tactical and operational levels, and the agent-based models (ABM), which is focused at the strategic level.

### Methods

Experimental research has long been employed by psychologists to study human behavior dating back to the Hawthorn Experiments of the 1930s, the controversial studies of authority found in the Mintzberg and Stanford Prison Experiments, and to a wide range of social psychological studies of human perception (24). As a result of these studies, a diverse and growing understanding of game theory, social cues and behavioral nudging has emerged and is informing all of the social sciences. The advancement of computer simulation and gaming software is now allowing researchers to employ gaming and simulation techniques to study human behavior across different scales of decision making. The use of games for research purposes dates back at least to the iterated prisoner's dilemma experiments of Robert Axelrod among others (35). The terms “serious gaming” and “online field experiments” (36) have been employed across the social sciences to study a diverse array of phenomena (37). The salient features of these games and experiments are the abilities to define initial contexts and settings, recruit a diverse array of participants, and employ a range of treatments to test hypotheses and tune theories.

While serious games are being used to study human behavior under specific contexts and treatments, object-oriented, agent-based models are now proliferating across the social sciences, veterinary sciences, including epidemiology. The introduction of different types of network node behavior into a class of object-oriented ABMs is now allowing for stochastic behaviors resulting from human agency to be aggregated to larger systems levels, permitting for the operational and tactical decision-making of agents to essentially “scale up” into larger, emergent system patterns. ABMs are models of dynamic systems that allow for the emergence of self-organizing properties found in complex adaptive systems. A number of ABMs that have been structured using network and graph theory have been advanced by public administration scholars (38, 39), providing for very fertile grounds to model and study network and collaborative governance structures (40). The ABM simulates agents designed to adapt to various simulated outbreak scenarios. After many iterations of the ABM, maps predicting likely outcomes with different agent perturbation can be produced (41).

The structures of the two games and versions of the ABM were developed over time in collaboration with a variety of experts in the field of livestock disease transmission. Our team first sought to ensure the simulations adequately represented the structure of swine production chains and how diseases are transmitted across networks of facilities. We drew on a series of focus groups and workshops with experts including veterinarians, industry association representatives, Extension agents, university researchers, policy makers, owners of whole production chains, suppliers and owners of production facilities to inform our understanding. These experts confirmed the types of biosecurity being enacted by swine producers and the problems with containing disease transmission. The design for the compliance game (emerged as these experts described how biosecurity is enacted in facilities and the challenges associated with maintaining front line workers' compliance with biosecurity protocols. We learned from them, for instance, of the propensity of workers to violate protocols to take “smoke breaks” or undertake time sensitive tasks. The idea for the protocol adoption game emerged during discussions with experts around the value of early warning systems relating to disease locations and biosecurity mapping tools to track who and where biosecurity protocols are enacted. They sought to learn from our games how best to communicate messages and whether to share information in real time with producers across infected production chains. The design of the ABM also emerged out of expert's descriptions of the production chains and the availability of data on the number and type of facilities within three different states.

What follows is a more detailed description of the components of the interrelated operational, tactical, strategic simulations used to explore the relation between micro and macro effects of decision making.

1. A Biosecurity Compliance Game (“Compliance Game”) that is designed to study *operational* decisions of “workers” in facilities to implement biosecurity measures or not. This game is particularly focused on how different risk communication treatments may impact a participant's (playing the role of farm employee) choice to follow established protocols. Our published results suggest that visual threat gauges are most effective at communicating the uncertainties associated with the disease threat environment, as opposed to traditional means such as numerically or linguistically presented risk information (22, 42).
2. A Biosecurity Protocol Adoption Game (“Protocol Game”) that is designed to simulate the *tactical* decision of managing biosecurity investment over time across several outbreak scenarios. This game is focused on the role that environmental and social uncertainty play in a “farmer's” willingness to adopt biosecurity measures. The availability of information about the disease prevalence, location and peer-behavior related to a disease in the system can either stimulate or inhibit biosecurity adoption in complex ways. For example, a disease that is known to be creeping spatially closer to a decision-maker's premises tends to encourage adoption behavior, whereas awareness of peers' biosecurity adoption decisions decreases adoption (23). Based on data from these games, we identified three major clusters of human behavioral strategies (43), differing based on risk preference.
3. A Regional US Hog Production Network Biosecurity Model (RUSH-PNBM) that is designed to provide strategic and tactical level understanding by simulating the flow of livestock across an empirically calibrated production chain network. This ABM simulates the transmission of diseases relative to the network structures, livestock flows, human behavior and experimentally-derived biosecurity adoption rates. One version of the model is calibrated for the swine industry in three states: Iowa, North Carolina and Illinois (30–32). The RUSH-PNBM is built to simulate transmission of disease among hog industry facilities through infected pigs and contaminated feed or other objects. Encoded disease drivers include farm visitors and trucks transporting hogs or feed possibly infected by the virus. Findings from the protocol adoption experimental game are used to inform agent decision heuristics in RUSH-PNBM (32). Data collected from game outputs inform the distribution of heterogeneity, the proportion of the agents programmed to behave based on a range of risk aversion and risk-taking behaviors observed in the protocol adoption game (23, 43, 44). Under the ongoing USDA NIFA project, a national level ABM is being designed and tested.

Table 1 provides an overview of the game or model component, the level at which they are framed, published studies sharing results of each specific game or model and the different behavioral theories that are cited to set up and discuss the results. The experimental simulation platform allows for the integrated study of human behavior at the operational and tactical levels by drawing on a range of behavioral theories. These behaviors, in turn, can be simulated percolating through production chain networks in an ABM providing for strategic level assessments. We now turn to a more detailed summary of specific application of each component of our approach and key findings.

**Table 1:** Experimental simulation platform publications and theoretical underpininngs.

**Game/ Model**	**Level**	**Studies**	**Behavioral theories**
Compliance game	Operational	Merrill et al. (22) Trinity et al. (45) Merrill et al. (42) Liu et al. (see footnote 1)	Theory of planned behavior (46) Prospect theory, Kahneman and Tversky (24) Discounting of future (28, 29) Diffusion of innovation theory (33) Information display (47–49) Uncertainty aversion (27) Cultural influences of uncertainty aversion (50)
Protocol Adoption Game	Tactical	Merrill et al. (23) Clark et al. (43). Clark et al. (51)	Prospect theory (24) Framing and communication effects (52) Uncertainty, Social and Environmental (53) Risk aversion and incentive effects (25, 26) Discounting of future (28, 29) Uncertainty aversion (27)
Agent-based model: Regional US Hog Production Network Biosecurity Model (RUSH-PNBM)	Strategic	Wiltshire (30) Wiltshire et al. (31) Bucini et al. (32)	Percolation theory (34, 54) Diffusion of innovation theory (33)

## Results

### Biosecurity protocol compliance game

**Figure 3:**
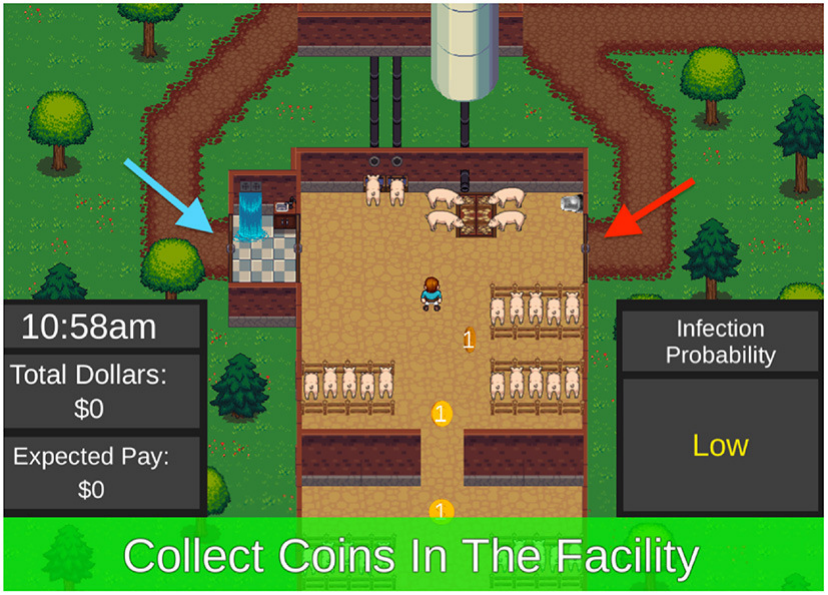
Compliance Game Facility Screenshot (22). Screen view of infection risk and uncertainty information for one game round, the worker and coins (internal tasks) inside the barn, the shower facility (blue arrow) and the emergency exit or “dirty door” (red arrow), and paths taken to attend to outside tasks. During each round participants collect coins within the barn (internal tasks), then receive a cue to complete a task outside (external task). The participants would then make decisions to use the shower-in, shower-out biosecurity practice or the emergency exit to complete the outside task based on the information provided. After completing the outside task the participant would return to the barn and collect more coins before ending the working day.

The Biosecurity Compliance Game focuses at the operational scale in which a worker is presented with a series of tasks to complete both within and outside of the barn (see screenshot in Figure 3). A “emergency exit” to the right and a “shower exit” to the left are options for entering and exiting the barn to undertake outside or external tasks such as unloading a truck or clearing a clogged feed line. Disease may enter the barn through contamination brought in by the player through the dirty door. Both internal and external tasks are awarded points. Points for external tasks decrease with time, leading to a heightened sense of urgency. Players receive a cash payout for performance. Time lags and economic costs are built into the clean door exit to present the player with a cost-benefit challenge between exiting the clean door vs. the dirty door. Various treatments—including messaging, increasing the likelihood of catching a disease and disease risk uncertainty—are implemented. The game attempts to simulate the types of decisions farm workers need to make on a daily basis. These are key operational decisions that center on the fidelity of the best management practice.

The major focus of the compliance game concerns the factors that encourage the routine practice of biosecurity protocols at the operational farm scale. The core questions include: What types of rewards and punishments are more or less likely to bring about greater compliance? How do time constraints and minute-level features of biosecurity procedures impact compliance rates? What kinds of messaging are most effective to bring participants into compliance? Different levels of messaging of infection risks are communicated linguistically (from very low to high), numerically (1%−25% chance of disease) and graphically [using a threat gauge pointing to values on a color-coded gauge ranging from very low (green) to high (red)]. Levels of uncertainty were depicted as either having no uncertainty, with a known value presented, or depicted as having uncertainty, with the values above and below presented as a possible range, as well as presenting a best estimate value (e.g., best estimate of 5%, with infection risk likely between 1 and 15%).

**Figure 4:**
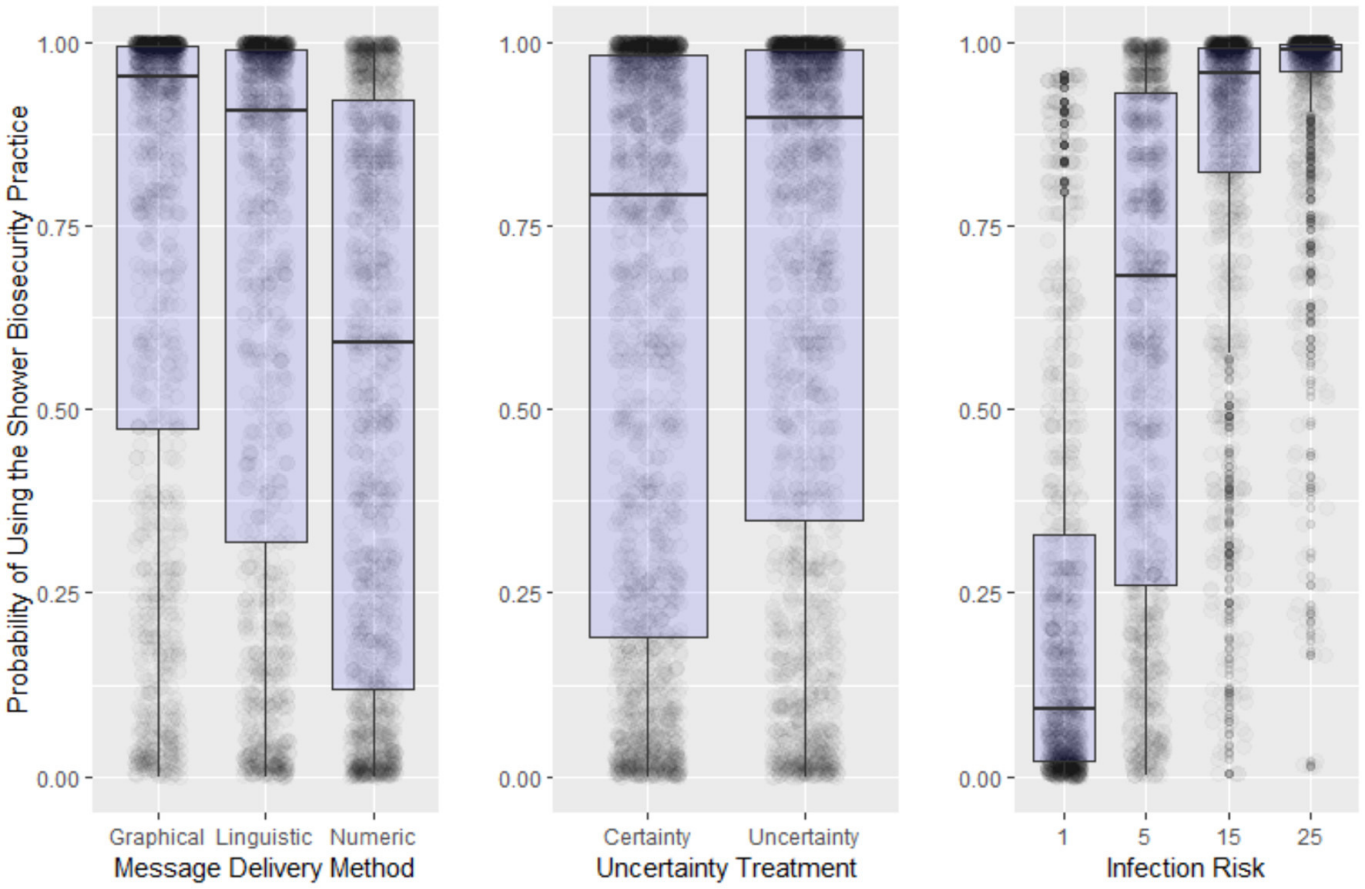
Summary results of the main treatment effects (22). Box-plot of the probability of using the shower biosecurity practice by the main effects, delivery method (*x*-axis) and infection risk (*y*-axis), and upper box boundaries 25 and 75th percentiles, respectively, line inside box median, overlaid on model predicted data values.

Although several papers have been published from this game using a variety of other combinations of treatments (22, 45), the specific results from an experiment testing different modalities of information display are displayed in Figure 4. Linguistic, numeric, and graphical messaging and higher threats of disease all lead to higher rates of compliance with biosecurity protocols at the operational level.

The stand-alone results from this particular experiment show that participants playing as front-line workers do perceive and take in risk messaging. Higher levels of communicated risk lead to higher levels of compliance with biosecurity protocols (far right panel of Figure 4). Another key finding from this particular experiment is that the mode of communication—linguistic, numeric or graphical, has a bearing on participant compliance with biosecurity protocols.

These findings also highlight the challenges that arise when compliance with organizational rules and protocols are called for. Prospect theory was used to explain why many participants chose to avoid doing the most biosecure practice because of asymmetric forms of risk aversion found among the population of participants (24). We also found that many participants tended to discount the potential of future losses (22), displaying evidence of temporal discounting theory (28, 29). These findings underscore the need for facility owners, managers and policy makers to consider the role that the heterogeneity of risk aversion and discounting effects play in shaping front line workers' actions. These considerations could be used to improve the efficacy of biosecurity adoption and incenting decisions at tactical and strategic scales. It should also be noted that cultural differences may influence how people respond—meaning that prospecting, discounting and free-riding behaviors are very likely influenced by cultural factors as well. A recent study undertaken by Lui et al.^1^, drew on the compliance game to study the role of cultural influences on risk assessment by offering both an English and Spanish language versions of the game. Hofstede's cultural uncertainty avoidance index (50) was used to frame a hypothesis relating to anticipated differences in risk avoidance and risk communications between Spanish and English-first language speakers. The findings from this study yieled mixed results, as we found no significance differences in risk aversion, but did find some differences in risk communication—as Spanish-first speakers tended to be influence less by graphical messaging than English-first speakers (Lui et al.^1^).

### Biosecurity protocol adoption game

**Figure 5:**
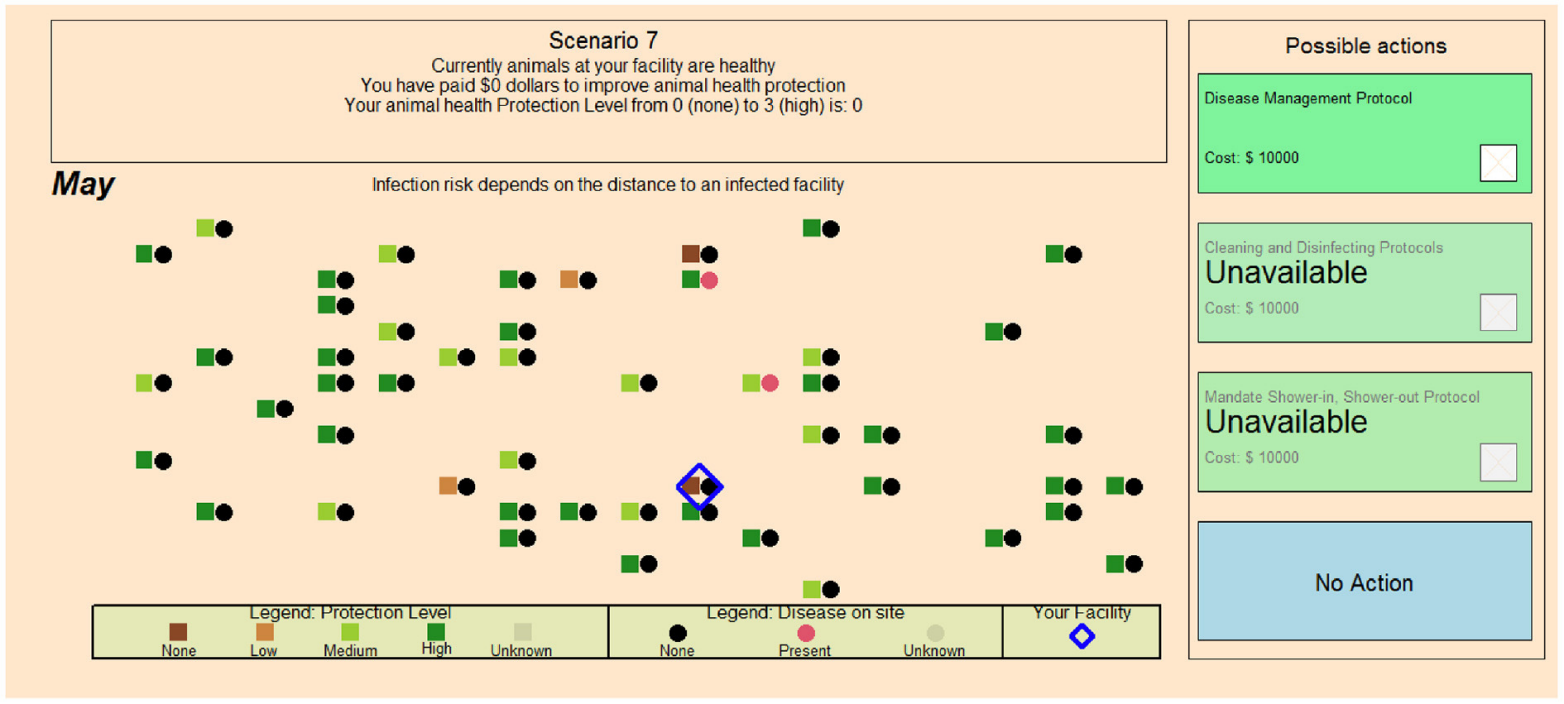
Protocol adoption game regional proximity of disease threat and biosecurity adoption (23).

The biosecurity protocol adoption game considers the tactical decisions of farm owners or managers. Figure 5 is a screen shot from one game that shows the field of play in a geophysical space that provides the participant with their farm's proximity to neighboring farms as a simple, geographically scale-free representation of the location of the participant's facility relative to neighboring ones. Information about disease spread is signaled by the circle symbol. When the circle is red, the farm has disease. Black signifies no disease, and gray signifies an unknown condition. The squares symbolize farm level adoption of biosecurity measures. A color scale ranging from brown to dark green symbolizes no to high levels of investment in biosecurity. Gray squares symbolize an unknown status. A set of possible actions (see right hand column) allows for the adoption of specific biosecurity protocol adoption actions. Each action comes at a cost that cuts into the farm's economic bottom line and therefore the participant's payout.

The protocol adoption game allows for the testing of typologies of behavior and information communication strategies by eliciting risk perceptions of participants assuming the role of facility managers who make the tactical decision of whether or not to adopt the kind of biosecurity protocols found in the compliance game. One of the major treatments simulated in the protocol adoption game is the alteration of levels of information sharing about disease prevalence and biosecurity investment levels of peers. Currently, limited information sharing or reporting is the industry norm, resulting in uncertainty as to the incidence of disease and uncertainty regarding the response to the threat of disease by other stakeholders in livestock industries. In this game, we altered disease incidence information sharing and biosecurity practice information sharing. Information levels were equated with environmental uncertainty (location of disease) and social uncertainty (levels of biosecurity adoption by neighbors) (23, 43).

The central questions that can be addressed through the analysis of this game's results include: What incentivizes producers to invest in biosecurity practices? How do perceptions of uncertainty regarding the location and prevalence of disease and of peers' adoption of biosecurity practices impact producer willingness to invest in biosecurity practices? How much and what type of information should be made available? Merrill et al. (23) have published results that demonstrated participant's willingness to invest in biosecurity when faced with treatments that varied the rates of information about disease incidence (described in the paper as “environmental uncertainty”) and information about the biosecurity response to simulated pork production facilities to the threat of disease (described in the paper as “social uncertainty”).

Findings from this experiment (see Table 2) suggest that willingness to invest in heightened biosecurity increases with increased awareness of disease incidence in the system, but decreases with increased awareness of biosecurity practices in place at nearby facilities (23). Results suggest that policy interventions incentivizing or mandating increased communication or information sharing could have positive or negative repercussions on the livestock industry's resilience to disease threats, depending upon the type of information shared.

**Table 2:** Results of protocol adoption game Kolmogorov–Smirnov tests (23).

	**Disease prevalence**	**Biosecurity adoption**
**No information vs Partial information**	*D* = 0.061717, *p* = 0.1621	*D* = 0.11515, *p* < 0.001
**No information vs Complete information**	*D* = 0.1663, *p* < 0.001	*D* = 0.12668, *p* < 0.001
**Partial information vs Complete information**	*D* = 0.11364, *p* < 0.001	*D* = 0.041298, *p* = 0.627

This study looks at risk uncertainty as a matter of no, partial, or complete information about disease risk and the levels of risk resulting from neighbors' biosecurity adoption rates. These findings suggest that having partial to complete information about the instances of disease within neighboring farms leads to more biosecurity adoption among participants. The threat of systemic disease risk is better visualized with partial to complete information. This treatment effect controls for actual disease threat. The mere exposure of disease risk as a concept embedded in the partial to fully known disease prevalence increases risk aversion across our sampled participants. These findings were confirmed in a second published study (43), which categorized behavioral strategies and participant risk profiles in response to simulated risk communication.

The same cannot be said about the levels of information regarding the prevalence of biosecurity practices of neighboring farms. Given no to partial information about peers' biosecurity adoption led participants to be more risk averse—leading to a increase in their choice to adopt biosecurity measures on their own farms. Again, these results control for simulated variance in adoption rates. This latter finding can be explained in one of two ways. The first is the free-rider effect (55) commonly referenced in economics. When someone else is shouldering the burden of protecting a network, there is a tendency for rent-seeking or free-riding mentality: Why should I have to pay for something that can be paid for by someone else? In the case of Merrill et al.'s findings (23), free riders would perceive that at least some neighbors are protecting others, and that is enough. To invest oneself is a sucker's bet. A second explanation may be that the participants simply do not understand how disease transmission spreads. The version of the protocol games used in this experiment did not present the neighboring farms as networks, connected by roads or supply chains (feed routes for instance). Participants had no way to clearly infer that disease risks of neighbors increases one's own risk of getting the disease. This potential misunderstanding can be tested in future iterations.

The choice to invest in biosecurity practices is based on the tactical risk assessment of the participant. This study demonstrates that the matter of when, if at all, to invest in biosecurity practices is at least in part shaped by the level of information participants had regarding systemic risk—the risk taking or aversion actions of neighbors, and the use of available knowledge about where, if at all, there is disease among neighboring farms. To explain the results from this game, prospect theory (24) and temporal discounting theory (28, 29) were also employed. These theories were used to explain why participants delayed or refrained from acting to mitigate risk as game play wore on. We also drew more on recent risk communication theories such as the role of framing and communication effects (52), and risk aversion and incentive effects (25, 26) to better understand and explain why participants responded to specific messaging and risk-reward inventives. These latter theories are extensions of prospect theory and provide deeper insights into the relationship between messaging and risk aversion. A final theory drew in the role that uncertainty and uncertainty aversion plays in making social and environmental decisions (27, 53).

These results can have very concrete implication for strategic leaders in industry and government. Industry leaders, like the owners of complete production chains or hubs in networks of contract farmers, can use these findings to consider the likelihood that other members of their production networks or within their geographic regions are more or less inclined to adoption biosecurity. While policy makers and government agencies may want to consider the role of public information campaigns, early warning systems and other information sharing tools regarding disease prevalence and location, and regarding the locations of facilities that have invested in biosecurity.

### RUSH-PNBM

**Figure 6:**
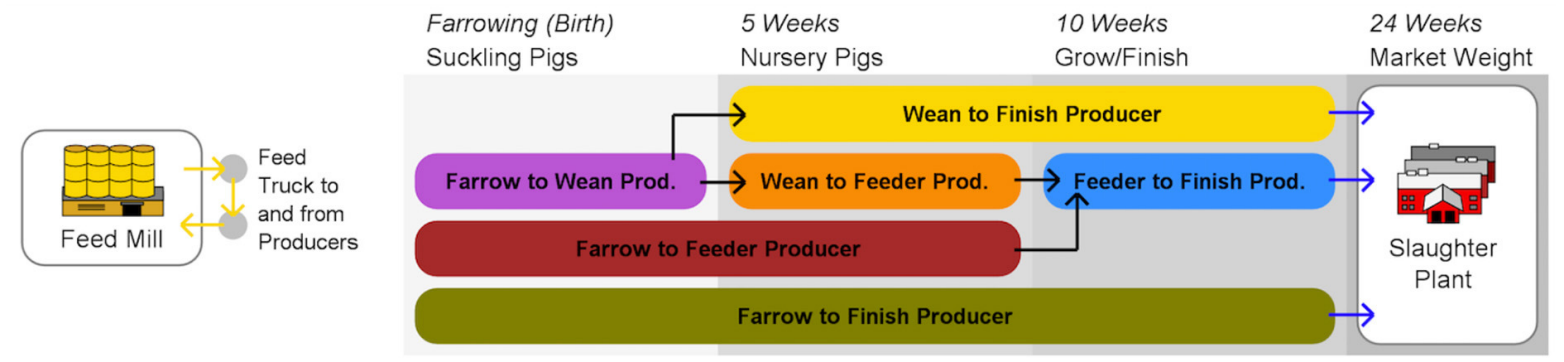
RUSH-PNBM agent connectivity key (30).

Following extensive engagement with industry stakeholders for data sourcing and acquisition, the modeling team set out to simulate key industry structures, functions and practices. Descriptive statistics of the breakdown of industry actors at the US county level were used to construct an ABM of the industry in three different states of the country. Figure 6 (below) depicts the major swine industry production actors in the United States identified as key actors in disease transmission, and represented in the RUSH-PNBM. These actors include farmers (or producers) focusing on certain stages (e.g., wean to finish) or all (farrow to finish) of a hog's lifecycle, feed mills and slaughter plants, as well as information providers represented by veterinarians. Some of the core questions that can be addressed by this ABM are: How does the structure of the production chain impact a facility's likelihood of getting a disease? Which components of the production chain are most vulnerable to disease threat? Where should investments in biosecurity measures be made?

**Figure 7:**
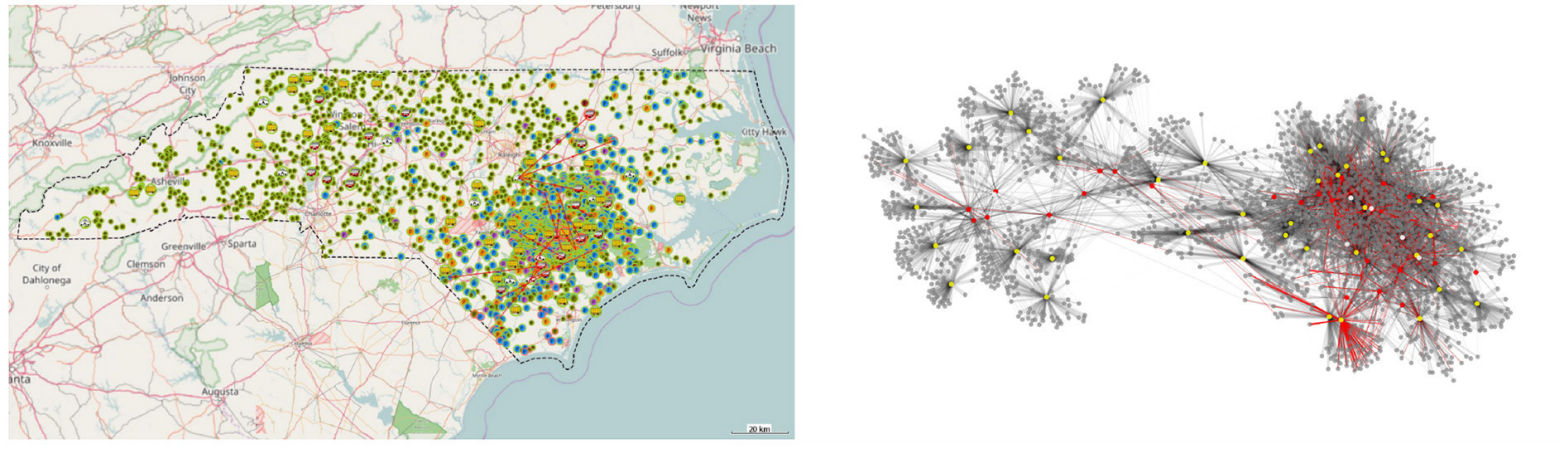
Example state level network configuration for North Carolina, as represented by RUSH-PNBM.

The RUSH-PNBM simulates disease transmission through the network using widely accepted Susceptible-Infected-Recovered-Susceptible (SIRS) model parameters. Distributions of tendencies or willingness to invest time or money into biosecurity as well as disease transmission parameters can be varied to generate different scenarios. The production chain network model embedded in the ABM provides a system view of the flow of swine and feed across a region (state level). For example, Figure 7, below, displays the configuration of facilities generated by RUSH-PNBM in a spatially approximate model of swine facilities for the state of North Carolina. The network graph to the right shows the agents' connection and disease transmission patterns over the course of one model run.

Computer simulations describe possible disease outbreaks scenarios driven by factors such as rate of spread, method of contact, probability of outbreak occurrence, and economic effect on both national and international markets. Additionally, we parameterize disease contagion as a function of biosecurity adoption. Building on existing ABM platforms devised by the gaming and simulation team (30, 56), “agents” within the ABM framework are programmed to mimic stakeholder profiles (i.e., to simulate heterogeneous producers, distributors, and, for more advanced games, regulators) and decision metrics. As the heuristic and decision metrics have been quantified using the protocol adoption game results, the ABM can simulate outbreaks (57) for each production type based on heterogeneous risk behavior. Because many of the processes, such as disease transimission, are stochastic within the ABM, thousands of model simulations can be run, each being unique, which generates a set of reasonable expectations for each set of assumed conditions, and thus allows for scenario planning. Table 3 lists the key model parameters of RUSH-PNBM.

**Table 3:** Model parameters and values used in the RUSH-PNBM model (31).

**Parameter**	**Value(s)**
Study area	[NC, IA, IL]
Number of producers in study area	[2,217, 6,266, 2,045]
Avg. producer capacity	[4,015, 3,265, 2,264]
Proportion farrow to wean	[0.050, 0.026, 0.038]
Proportion farrow to feeder	[0.005, 0.010, 0.009]
Proportion farrow to finish	[0.554, 0.304, 0.635]
Proportion wean to feeder	[0.102, 0.064, 0.023]
Proportion wean to finish	[0.003, 0.077, 0.055]
Proportion feeder to finish	[0.286, 0.519, 0.241]
Number of slaughter plants in study area	[24, 18, 25]
Number of feed mills in study area	[40, 114, 37]
Producer to slaughter plant λ	2
Producer to feed mill λ	1.5
Percent of producers initially infected	5%
Avg. producer infection duration (days)	40
Avg. slaughter plant infection duration (days)	7
Avg. feed mill infection duration (days)	25
Suckling pig mortality rate on infection	0.9
Nursery pig mortality rate on infection	0.4
Grow/finish hog mortality rate on infection	0.1
Prob. producer will become infected if returning pig truck is contaminated	0.3
Prob. producer will become infected if feed truck is contaminated	0.8
Prob. feed truck will become contaminated if producer is infected	0.05
Prob. pig truck will become contaminated if producer is infected	0.2
Prob. feed mill will become infected if returning feed truck is contaminated	0.1
Prob. feed truck will become contaminated if feed mill is infected	0.5
Prob. slaughter plant receiving area will become infected if pig batch is infected	0.4
Prob. pig truck will become contaminated if receiving area is infected	0.2
Farrow to wean sow proportion (relative to total capacity)	0.6
Farrow to feeder sow proportion (relative to total capacity)	0.5
Farrow to finish sow proportion (relative to total capacity)	0.2
Annual number of piglets per sow	34
Max. wean and batch frequency (days)	7
Min. batch size (as proportion of non-sow capacity)	0.05
Capacity under which producer has only one batch	20
Min. capacity similarity ratio	25
Max. producer to producer shipment distance (km)	150
Max. number of potential producer to producer trading partners	15
Max. producer to producer shipment frequency (days)	5
Avg. number of daily feed delivery trips per mill	10
Number of producers visited per feed delivery trip λ	1

“EAP” indicates that the value was derived through expert advisory panel sessions. “FHPC” refers to data provided to us by commercially-owned hog production chain systems.

In his paper evaluating the effect of producer specialization on the epidemiological resilience of livestock production networks, Wiltshire (30) looks at the structure of the hog industry at regional scales and runs a series of experiments changing the specialization structures of the industry as follows: (1) high specialization three-phase production systems (equal numbers of Farrow to Wean, Wean to Feeder, and Feeder to Finish producers); (2) medium specialization production systems (equal numbers of Farrow to Feeder and Feeder to Finish producers); and (3) low specialization production systems (Farrow to Finish producers only).

Drawing on percolation theory (34, 54, 58), Wiltshire finds that multi-phase (e.g., more complex) production systems are vulnerable to catastrophic outbreaks at lower spatial densities, have more abrupt percolation transitions, and are characterized by less predictable outbreak scope and durations. Following other multi-phase network studies, we suggest that the absence of potentially-bridging producer-to-producer edges is largely responsible for the superior disease resilience of single-phase production systems. The results of this experiment strongly suggest that, at least in the context of this model, the risk of catastrophic infectious disease outbreaks may be inhibited by (a) sparser producer networks, and, perhaps more critically, (b) networks in which fewer contacts for interaction facilitate greater compartmentalization of inter-agent contact patterns, leading to both shorter and smaller outbreaks, as well as less uncertainty about the final size and duration of a given outbreak. Both these findings have implications for strategic risk management in livestock production chain systems.

**Figure 8:**
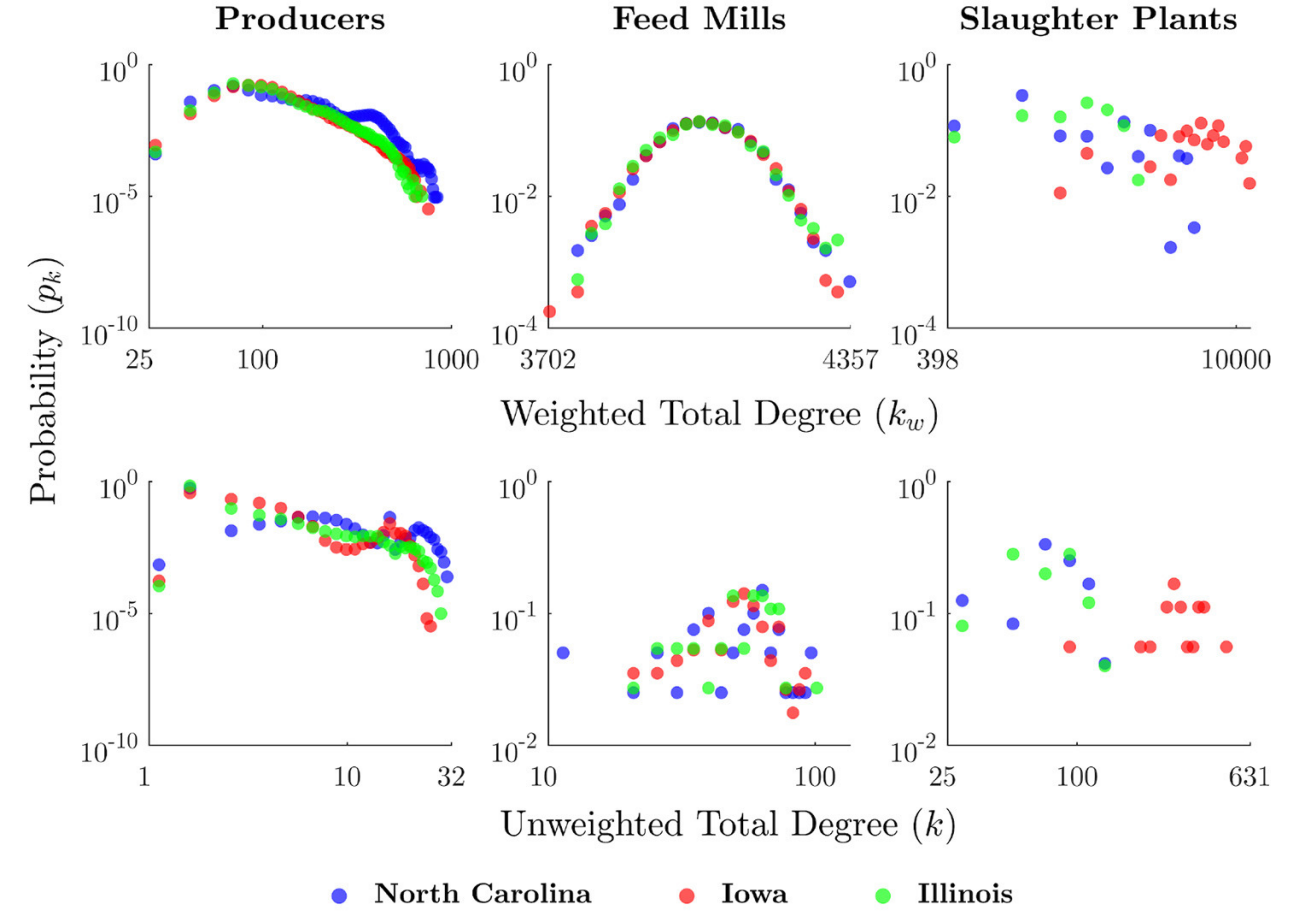
Degree plots for RUSH-PNBM experiment (31). Weighted degree (kw) represents the number of times an agent contacted another agent (i.e., sending or receiving either livestock or feed) over the course of a run, whereas unweighted degree (*k*) describes the number of unique agents with which an agent had contact.

In Wiltshire et al. (31), RUSH-PNBM is used to examine in greater depth the role of network structure and agents' positions within contact networks on disease resilience of the modeled system. A series of disease spread scenarios are run through independent swine production chain networks in three different high production states: North Carolina, Iowa and Illinois. Figure 8 shows some results from this network analysis, indicating the general connectivity patterns of each actor type, and how they differ between the three different states.

This analysis demonstrates the model's capacity to simulate disease transmission over distributed networks of heterogeneous agents. The structure of the production chain both constrains and enables disease transmission. The function of different facilities as positions in these value chain networks have a bearing on a type of facility's propensity to transmit or receive disease.

**Figure 9:**
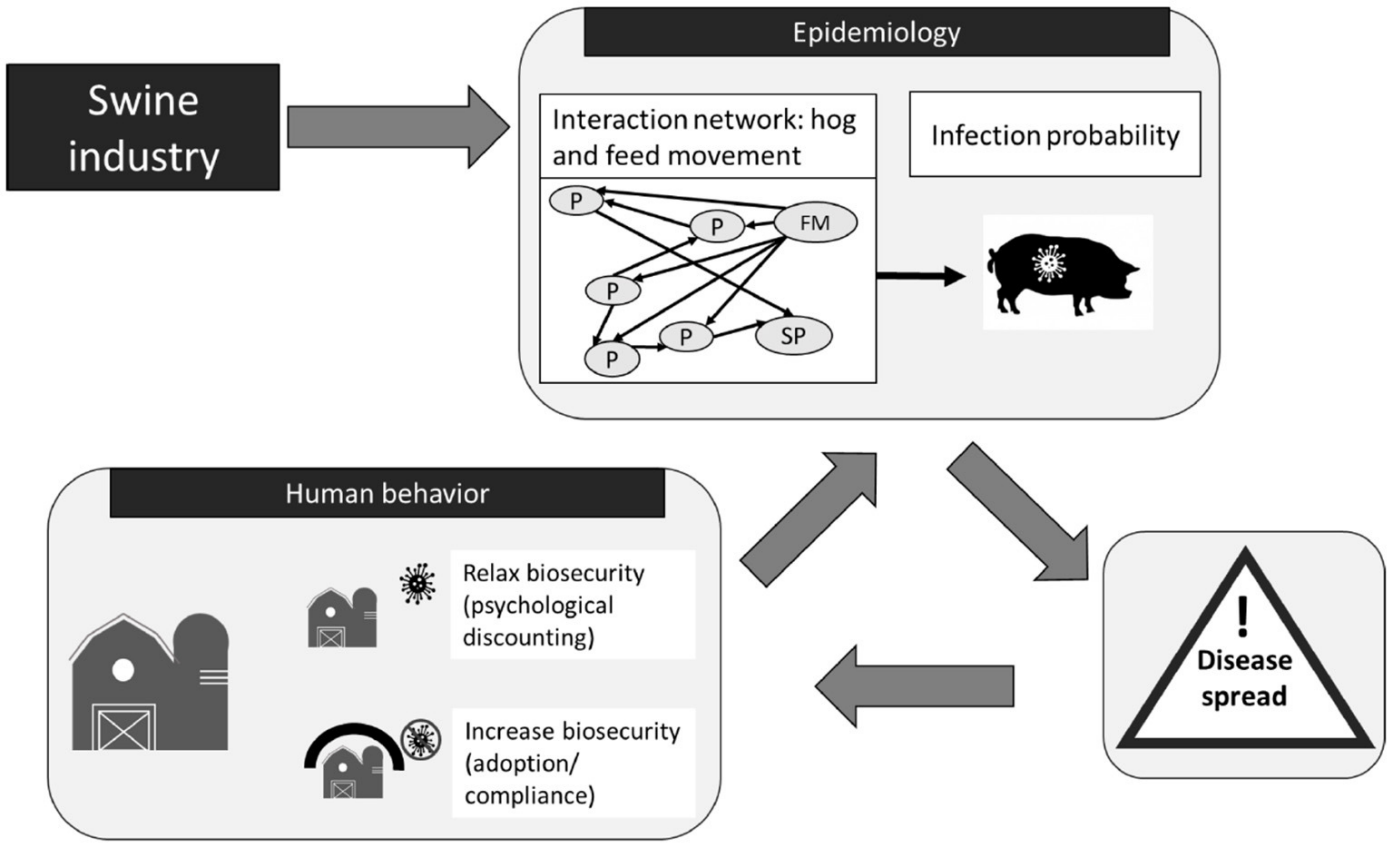
RUSH-PNBM Agent-based model (ABM) process flow (32) that highlights the ABM's main components and processes of how PEDv can spread through the network structure of the swine industry and influenced by human risk behavior. Types of agents in: P, producer; FM, feed mill, and SP slaughter plant. Hog and feed movement are simulated. The epidemiological component simulates risk of PEDv transmission associated with movement through networks. Human decisions on biosecurity also influence infection risk. Disease spread depends on the probability of disease transmission in the networks and influences the biosecurity levels on farm.

An evolved version of the RUSH-PNBM model simulated at a county level adds an additional behavioral signal, “risk attitude” (32). In these instances, agents in the model could implement certain levels of biosecurity. The behavioral signal is the simulated facility agent's propensity to adopt biosecurity or not, given the disease conditions in the systems. Figure 9 displays a high-level visualization of the main features of the RUSH-PNBM component parts, including an Susceptible-Infected-Susceptible (SIS) disease spread model and the human behavioral signal of the decision to adopt biosecurity.

An experiment was conducted that varied the relative proportion of risk-averse vs. risk-tolerant producers (32) using the risk attitudes drawn from the Protocol Adoption Game results highlighted above (23). Agents display clustered behavior along a risk-tolerant to risk-averse behavioral spectrum that was correlated within the ABM with the probability or propensity to make the decision to implement biosecurity or not (43). To reflect this heterogeneity, agents in the model are imbued with agent decision making processes that include parameters for risk attitude, responsiveness to disease, biosecurity investment and psychological distancing. The key parameter, risk-attitude, drives the agent's biosecurity investment as a response to the number of infected neighboring facility agents.

**Figure 10:**
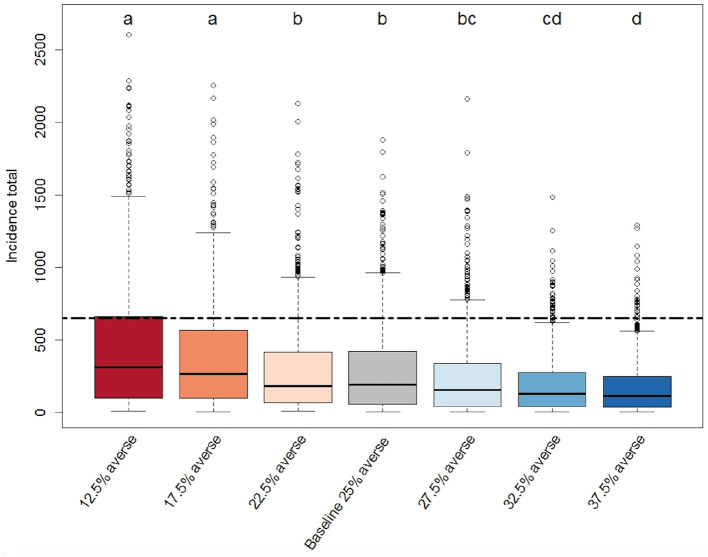
Box-plot of the distribution of total PEDv incidence (sum of new infection cases over the simulated time period) for each scenario (32). Each scenario represents a different distribution of risk attitude within the population of producer agents in the ABM. The baseline scenarios population has equal proportion of producer agents in all four groups (risk adverse, risk opportunistic risk neutral, and risk tolerant). The three left hand scenarios tested the effect of reducing the number of risk averse agents (by 10, 30, or 50%, respectively) and shifting them to risk tolerant. The three right hand scenarios shifted risk tolerant (by 10, 30, and 50%, respectively) to risk averse. Each scenario distribution is drawn from a Monte Carlo experiment with 800 replicates. The compact letter display indicates significance from pairwise comparison. The black dashed line marks the total incidence in the observed data.

Results show that the system's epidemiological resilience is sensitive to fairly minor changes to the risk attitude parameter. Figure 10 demonstrates how the percentage of facility managers in the model displaying risk averse behaviors among a range of 12%−37.5% risk averse affect disease incidence in the system. There is a significant difference in median disease incidence between the “baseline” (25% risk averse) scenario runs and the “27.5% risk averse” scenario runs, which include just 10% more risk-averse producers than the baseline. The authors conclude that, “Overall, the scenarios indicate that the ABM is significantly sensitive to risk attitude shifts as small as 10% producer agents moving from being risk tolerant to being risk averse” [(32), p. 6].

Bucini et al. (32) also find that increasing the number of risk-tolerant producers in the system leads to more variability in epidemic severity, supporting the percolation dynamics reported in Wiltshire (30), and suggesting that the addition of risk- tolerant producers to a production system could hamper the efforts of policymakers who rely on disease forecasting to guide reactive decision-making in the face of an epidemic threat. The correlation between increased risk tolerant decision makers at the tactical level and higher variability in disease impact means that, with high proportions of risk tolerant individuals, emergent diseases could either quickly disappear (which is consistent with high proportions of risk averse decision makers) or rapidly reach epidemic levels. That is, the model shows how risk tolerant populations (who have not paid associated costs for biosecurity), based on the distribution of probabilistic events avoid significant impacts or, in essence, get lucky. Yet, the model shows that, on average, strategically nudging or shoving populations toward more risk averse tendencies will reduce the impact of emergent diseases on the production system.

The strategic implications of the findings for experiments run across the different versions of the RUSH-PNB are very clear: the variabilities and stochasticity of human behaviors, as noted in the probability distributions of risk takers and risk adverse agents plays a significant factor in the spread of disease, further bolstering the need for stronger incentives and regulations that can be used to overcome a preponderance of defectors or resisters to biosecurity.

## Discussion

The platform of companion games and models presented here represent a multi-level, multi-scale approach to modeling a complex adaptive system that includes individuals (front line workers, facility owners, production chain owners), organizations (facilities), and networks of organizations carrying out a heterogeneity of functions. The compliance game is able to capture the operational characteristics of the social system. We also rendered a comparative analysis of cultural differences between Enlish and Spanish-first language speakers and found that cultural differences can be factors [Lui et al. (see footnote 1)], leading us to assert that the findings discussed here should be taken within the dominant, North American, context within which most participants haled from. The protocol adoption game functions at the tactical scale and allows for understanding the decision to enact biosecurity or not. A model of the risk attitude of facility owners was developed from the protocol adoption game results and used to shape the probability distributions of risk attitudes within the ABM network. These findings were later validated by comparing Amazon Mechanical Turker results to those of industry stakeholders (43). The conclusion stemming from Bucini et al.'s work is one that stands to be of vital importance for the mitigation of disease transmission in livestock systems (and arguably for disease transmission among humans as well): that the risk attitudes of decision makers operating across a given production network will constrain or enable the spread of disease across the system. In other words, human behavior is a critical driver of disease spread. Micro-level decisions and behaviors of actors across the operational, tactical and strategic levels of an organization and wider system to adopt and/or comply with biosecurity protocols scale-up to form larger macro-level patterns. In the case of this example these patterns manifest themselves in the form of the spread of animal disease across a production system.

Although the papers synthesized here are limited on many fronts, including a narraow cultural context, we demonstrate the ability of our platform to model and experiment with different configurations of “psychological nudges” and “policy shoves,” (43) and examine how these interventions impact disease transmission. The range of psychological nudges tested across this platform include the use of different forms of messaging, information of risk levels, disease prevalence and biosecurity adoption rates of neighbors (22, 23, 43, 44, 51) [Lui et al. (see footnote 1)] including the use of “bots” to test efficacy of social cues (45). As in the real world, the participants in both games were rewarded for not employing biosecurity if they were lucky enough to not get the disease in their facility. In the case of operational, front-line workers, these rewards came in the form of additional time to complete tasks. In the case of tactical managers, the reward was saving money in the short term by not implementing biosecurity protocols. Both rewards were balanced against the risk of economic loss resulting from getting the disease on your premise. These kinds of trade-offs are increasingly a common feature in the development of biosecurity protocols and policies.

The ability of this platform to integrate different scales of social systems provides a replicable approach to modeling a complex network that includes both micro and macro level properties. Our approach to designing this experimental simulation platform allows for researchers to “nest” smaller questions of individual behavior and decision making within wider and bigger questions pertaining to whole system and network level patterns. Specifically, these smaller questions focus on how best to communicate risk and incentivize biosecurity to front-line workers (22, 45) (Lui et al.^1^) and facility owners and managers (23, 43). We demonstrate how the risk attitudes of facility owners and managers can scale-up to either increase or decrease the disease risk of an entire production chain. We are able to provide insight into matters of (micro)management and motivation and how these managerial matters scale-up to impact the performance of the wider system. In the case of our example here, this performance focuses on the ability of individual facilities and whole production chains to avoid or mitigate disease transmission.

Future iterations of the experimental simulation platform will include specific policy scenarios that are emerging from ongoing engagement with federal and state livestock animal health officials. In addition, we will test the compliance game using different languages. Questions such as: How would changes in indemnification programs through which the federal government covers the cost of loss of herds culled during disease outbreaks, impact biosecurity adoption rates? and Should more visible and spatially explicit disease outbreak and biosecurity adoption data be enacted? Implications of producer behavioral responses on reforming US federal policy of unconditional indemnity to cattle producers is also being investigated in the new USDA NIFA project. Dynamic integration of micro-macro scaling in spatially distributed ABMs may also provide context specific situational awareness to regulators and policymakers considering policy interventions in operational, tactical, and strategic policy making arenas.

## Conclusion

The suite of games and simulations designed and implemented to study PEDV in swine provides a unique set of insights into the real world complexities faced by livestock producers and service providers. Getting workers to comply, adopt, and consider a range of policy interventions to stem the tide of livestock disease spread unfolds within complex adaptive systems. The ability of researchers to consider the challenges associated with different scales of decision-making and their impacts on macro, systems level disease spread are only now just being understood. The ability of researchers and theorists to conceive of and execute multi-level, multi-scale studies and simulations requires deep engagement with stakeholders and transdisciplinary approaches.

At some point voluntary compliance at a scale needed to preserve a common pool resource or public good may not be possible (as the recent history with COVID-19 compliance bears out), as the propensity to defect or ignore may be reinforced by perceptions of short-term pay-outs or other factors such as ideological dispositions and trust of instititions. Simulation tools such as ABMs can be utilized to inform the emergence of systemic risk under different competing and cooperative strategies of players at operational, tactical and strategic scale. Equally, the resources needed to properly incentivize behaviors may not be available. A better understanding of the thresholds of when and where to use regulatory tools are needed.

## Data availability statement

The raw data supporting the conclusions of this article will be made available by the authors, without undue reservation.

## Ethics statement

The studies involving human participants were reviewed and approved by University of Vermont Research Protections Office. The patients/participants provided their written informed consent to participate in this study.

## Author contributions

All authors listed have made a substantial, direct, and intellectual contribution to the work and approved it for publication.

## Funding

This material is based upon work that was supported by the National Institute of Food and Agriculture, U.S. Department of Agriculture, under award numbers 2015-69004-23273, 2021-67015-35236 and 2022-69014-37041. Any opinions, findings, conclusions, or recommendations expressed in this publication are those of the author(s) and do not necessarily reflect the view of the U.S. Department of Agriculture.

## Conflict of interest

The authors declare that the research was conducted in the absence of any commercial or financial relationships that could be construed as a potential conflict of interest.

## Publisher's note

All claims expressed in this article are solely those of the authors and do not necessarily represent those of their affiliated organizations, or those of the publisher, the editors and the reviewers. Any product that may be evaluated in this article, or claim that may be made by its manufacturer, is not guaranteed or endorsed by the publisher.

## Footnotes

1 Liu et al. (under review). Effects of message delivery on cross-cultural biosecurity compliance: insights from experimental simulations.

## References

[1] Battaglio RP Jr Belardinelli P Bellé N. Behavioral public administration ad fontes: a synthesis of research on bounded rationality, cognitive biases, and nudging in public organizations. Public Adm Rev. (2019) 79:304–20. 10.1111/puar.12994

[2] Thaler RH Sunstein CR. Nudge: Improving Decisions about Health, Wealth, and Happiness. New York, NY: Penguin (2009).

[3] Moynihan D. A great schism approaching? Towards a micro and macro public administration. J Behav Public Adm. (2018) 1:1–8. 10.30636/jbpa.11.15

[4] Collins R. Theoretical Sociology. San Diego, CA: Harcourt College Pub (1988).

[5] Page S Miller JH. Complex Adaptive Systems: An Introduction to Computational Models of Social Life. Princeton, NJ: Princeton University Press (2009).

[6] Gulick L. Notes on the theory of organization. In:Shafritz J Hyde AC Parkes SJ editors. Classics of Public Administration, 5th ed. Belmont, CA: Wadsworth/Thomson Learning (1937, 2004). p. 90–8.

[7] Mintzberg H. Power In and Around Organizations. Englewood, Cliffs, NJ: Prentice-Hall (1983).

[8] Van Wart M. Dynamics of Leadership in public Service: Theory and Practice. Armonk, New York: M.E. Sharpe (2005).

[9] Scott RW. Organizations: Rational, Natural, and Open Systems, 2nd ed. Englewood Cliffs, NJ: Prentice-Hall (1987).

[10] Lipsky M. Street-level bureaucracy: The critical role of street-level bureaucrats. In:Shafritz J Hyde AC Parkes SJ editors. Classics of Public Administration. 5th ed. Belmont, CA: Wadsworth/Thomson Learning (2004). p. 414–22.

[11] Schein EH. Organizational Culture and Leadership, Vol. 2. Hoboken, NJ: John Wiley & Sons (2010).

[12] Mintzberg H. Mintzberg on Management: Inside Our Strange World of Organizations. New York, NY: Simon and Schuster (1989). 10.1007/978-1-349-20317-8_23

[13] Ackoff RL. Redesigning the future. Syst Pract. (1990) 3:521–4. 10.1007/BF01059636

[14] Loorbach D. Transition management for sustainable development: a prescriptive, complexity-based governance framework. Governance. (2010) 23:161–83. 10.1111/j.1468-0491.2009.01471.x

[15] White G. Strategic, tactical, and operational management security model. J Comput Inf Syst. (2009) 49:71–5.

[16] Ramos S Maclachlan M Melton A. A report from the economic research service a report from the economic research service impacts of the 2014-2015 highly pathogenic avian influenza outbreak on the U.S. Poultry Sector. (2017), p. 282–4. Available online at: www.ers.usda.gov (accessed May 16, 2020).

[17] Animal Plant Health Inspection Service (APHIS). Conformations of highly pathogenic avian influenza in commercial and backyard flocks. (2022). Available online at: https://www.aphis.usda.gov/aphis/ourfocus/animalhealth/animal-disease-information/avian/avian-influenza/hpai-2022/2022-hpai-commercial-backyard-flocks (accessed June 3, 2022).

[18] United States Geological Survey (USGS). Avian Influenza. (2019). Available online at: https://www.usgs.gov/centers/nwhc/science/avian-influenza?qt-science_center_objects=0#qt-science_center_objects (accessed January 15, 2020).

[19] Sumner J Ross T Jenson I Pointon A. A risk microbiological profile of the Australian red meat industry: Risk ratings of hazard-product pairings. Int J Food Microbiol. (2005) 105:221–32. 10.1016/j.ijfoodmicro.2005.03.0166 16099063

[20] Council for Agricultural Science Technology (CAST). Global Risks of Infectious Animal Disease. (2005). Available online at: https://www.cast-science.org/wp-content/uploads/2018/12/CAST-Global-Risks-of-Infectious-Animal-Diseases-Issue-Paper-28-FINAL69.pdf (accessed June 3, 2022).

[21] Schulz LL Tonsor GT. Assessment of the economic impacts of porcine epidemic diarrhea virus in the United States. J Anim Sci. (2015) 93:5111–8. 10.2527/jas.2015-9136 26641031 PMC7109690

[22] Merrill S Moegenburg S Koliba C Zia A Trinity L Bucini G. Willing to comply with biosecurity in livestock facilities: evidence from experimental simulations. Front Vet Sci. (2019) 4:156. 10.3389/fvets.2019.00156 31214603 PMC6558082

[23] Merrill S Koliba C Moegenburg S Zia A Parker J Sellnow T. Decision-making in livestock biosecurity practices amidst environmental and social uncertainty: evidence from an experimental game. PLOS ONE. (2019) 14:e0214500. 10.1371/journal.pone.0214500 30995253 PMC6469775

[24] Kahneman D. Tversky A. Prospect theory: an analysis of decision under risk. In:MacLean LC Ziemba WT editors. Handbook of the Fundamentals of Financial Decision Making: Part I. London: World Scientific (2013). p. 99–127. 10.1142/9789814417358_0006

[25] Holt CA Laury SK. Risk aversion and incentive effects. Am Econ Rev. (2002) 92:1644–55. 10.1257/000282802762024700

[26] Harrison GW Johnson E McInnes MM Rutstrom EE. Risk aversion and incentive effects: comment. Am Econ Rev. (2005) 95:897–901. 10.1257/0002828054201378

[27] Chakravarty S Roy J. Recursive expected utility and the separation of attitudes towards risk and ambiguity: an experimental study. Theory Decis. (2009) 66:199–228. 10.1007/s11238-008-9112-4

[28] Yi R Gatchalian KM Bickel WK. Discounting of past outcomes. Exp Clin Psychopharmacol. (2006) 14:311–7. 10.1037/1064-1297.14.3.311 16893274

[29] Caruso EM Gilbert DT Wilson TDA. wrinkle in time – asymmetric valuation of past and future events. Psychol Sci. (2008) 19:796–801. 10.1111/j.1467-9280.2008.02159.x 18816287

[30] Wiltshire SW. Using an agent-based model to evaluate the effect of producer specialization on the epidemiological resilience of livestock production networks. PLOS ONE. (2018) 13:e0194013. 10.1371/journal.pone.0194013 29522574 PMC5844541

[31] Wiltshire S Zia A Koliba C Buccini G Clark E Merrill S. Network meta-metrics: identifying effective indicators of epidemiological vulnerability in a livestock production system model. J Artif Soc Soc Simul. (2019) 22:8. 10.18564/jasss.3991

[32] Bucini G Merrill SM Clark E Moegenburg S Zia A Koliba C. (2019). Risk attitudes affect livestock biosecurity decisions with ramifications for disease control in a simulated production system. Front Vet Sci. 6:196. 10.3389/fvets.2019.00196 31294037 PMC6604760

[33] Warner KE. Need for some innovative concepts of innovation: an examination of research on the diffusion of innovations. Policy Sci. (1974) 5:433–51. 10.1007/BF00147229

[34] Stauffer D Aharony A. Introduction to Percolation Theory. New York: Taylor & Francis (2018).

[35] Axelrod R. Effective choice in the prisoner's dilemma. J Conflict Resolut. (1980) 24:3–25. 10.1177/002200278002400101

[36] Parigi P Santana JJ Cook KS. Online field experiments: studying social interactions in context. Soc Psychol Q. (2017) 80:1–19. 10.1177/0190272516680842

[37] Crookall D. Serious games, debriefing, and simulation/gaming as a discipline. Simul Gaming. (2010) 41:898–920. 10.1177/1046878110390784

[38] Maroulis S. Interpreting school choice treatment effects: results and implications from computational experiments. J Artif Soc Soc Simul. (2016) 19:7. 10.18564/jasss.3002

[39] Scott TA Thomas CW Magallanes JM. Convening for consensus: simulating stakeholder agreement in collaborative governance processes under different network conditions. J Public Adm Res Theory. (2018) 29:32–49. 10.1093/jopart/muy053

[40] Koliba C Zia A Merrill S. Using agent-based models to study network and collaborative governance. In:Voets J Keast R Koliba C editors. Researching Networks and Collaboration in the Public Sector: A Guide to Approaches, Methodologies, and Analytics. New York, NY: Routledge (2019). p.210–31. 10.4324/9781315544939-11

[41] Bone C Johnson B Nielsen-Pincus M Sproles E Bolte J. A temporal variant-invariant validation approach for agent-based models of landscape dynamics. Trans GIS. (2014) 18:182. 10.1111/tgis.12016

[42] Merrill SC Trinity L Clark E Shrum T Koliba CJ Zia A. Message delivery strategy influences willingness to comply with biosecurity. Front Vet Sci. (2021) 8:667265. 10.3389/fvets.2021.667265 34250060 PMC8269999

[43] Clark E Merrill SC Trinity L Bucini G Cheney N Langle-Chimal O. Using experimental gaming simulations to elicit risk mitigation behavioral strategies for agricultural disease management. PLoS ONE. (2020) 15:e0228983. 10.1371/journal.pone.0228983 32182247 PMC7077803

[44] Merrill SC Bucini G Clark EM Koliba CJ Trinity L Zia A. Why we need to account for human behavior and decision-making to effectively model the non-linear dynamics of livestock disease. In: Proceedings of the International Crisis and Risk Communication Conference, Vol. 4. Orlando Fl: Nicholson School of Communication and Media (2021), p. 23–28. 10.30658/icrcc.2021.06

[45] Trinity L Merrill SC Clark E Bucini G Koliba C Zia A. Effects of social cues on biosecurity compliance in livestock facilities: evidence from experimental simulations. Front Vet Sci. (2020) 7:130. 10.3389/fvets.2020.00130 32292792 PMC7120031

[46] Ajzen I. The theory of planned behavior. Organ Behav Hum Decis Process. (1991) 50:179–211. 10.1016/0749-5978(91)90020-T

[47] Patt AG Schrag DP. Using specific language to describe risk and probability. Clim Change. (2003) 61:17–30. 10.1023/A:1026314523443

[48] Visschers VHM Meertens RM Passchier WWF de Vries NNK. Probability information in risk communication: a review of the research literature. Risk Anal. (2009) 29:267–87. 10.1111/j.1539-6924.2008.01137.x 19000070

[49] Lipkus IM. Numeric, verbal, and visual formats of conveying health risk: suggested best practices and future recommendations. Med Decis Making. (2007) 27:696–713. 10.1177/0272989X07307271 17873259

[50] Hofstede G. Culture's Consequences: Comparing Values, Behaviors, Institutions and Organizations Across Nations. New York, NY: SAGE Publications (2001).

[51] Clark E Merrill SC Trinity L Bucini G Cheney N Langle-Chimal O. Emulating agricultural disease management: comparing risk preferences between industry professionals and online participants using experimental gaming simulations and paired lottery choice surveys. Front Vet Sci. (2021) 7:556668. 10.3389/fvets.2020.556668 33537351 PMC7848213

[52] Messick DM Allison ST Samuelson CD. Framing and communication effects on group members responses to environmental and social uncertainty. In:Maital SL editor. Applied Behavioral Economics. New York, NY: New York University Press (1988), p. 677–700.

[53] Suleiman R Rapoport A. Environmental and social uncertainty in single-trial resource dilemmas. Acta Psychol. (1988) 68:99–112. 10.1016/0001-6918(88)90048-0

[54] Son SW Bizhani G Christensen C Grassberger P. Percolation theory on interdependent networks based on epidemic spreading. Europhys Lett. (2012) 97:16006. 10.1209/0295-5075/97/16006

[55] Grossman SJ Hart OD. Takeover bids, the free-rider problem, and the theory of the corporation. Bell J Econ. (1980) 42–64. 10.2307/3003400

[56] Zia A Koliba C. The emergence of attractors under multi-level institutional designs: agent-based modeling of intergovernmental decision making for funding transportation projects. AI Soc. (2013) 30:315–331. 10.1007/s00146-013-0527-2

[57] Evans TP Manson S. Space, complexity, and agent-based modeling. Environ Plan B: Design. (2007) 34:196–9. 10.1068/b3402ed

[58] Tilman D Kareiva P editors. Spatial Ecology: The Role of Space in Population Dynamics and Interspecific Interactions (MPB-30). Princeton, NJ: Princeton University Press (1997). 10.1515/9780691188362

